# Advances in Genetics and Genomics of Animal Breeding: Call for Papers

**DOI:** 10.1093/g3journal/jkag132

**Published:** 2026-06-03

The journals of the Genetics Society of America, GENETICS and G3: Genes|Genomes|Genetics, are calling for submissions of papers on Advances in Genetics and Genomics of Animal Breeding.

This series will highlight innovative approaches, emerging strategies, and state-of-the-art methodologies shaping the future of farmed and companion animal breeding.

Specifically, we invite high-quality, original research and comprehensive reviews with a focus on:

- Cutting-edge genomic selection/prediction approaches that achieve demonstrable benefits in animal breeding, including layering of multiple omics data types and the use of large language models.
- Genome and phenome integration to identify gene-trait associations and functionally enriched genomic variation.
- Functional genomics approaches that leverage long-read and novel sequencing technologies to identify trait-linked variation at single-cell or bulk resolution.
- Development and application of *in vitro* and *in silico* models for functional validation of trait-linked variation.
- Precision breeding, digital twins, and high-throughput phenotyping with relevant implementation benefits.
- Pangenome and comparative genomics approaches that benefit animal breeding.
- Novel approaches for conservation of genomic diversity in managed populations.
- Ethical, societal, and sustainability considerations in modern breeding.
- New resources, tools, and approaches that enable the FAIR sharing of relevant data and metadata types.

This series welcomes advances in genetics and genomics, including novel technologies that may revolutionize animal breeding, enabling more precise, efficient, and sustainable improvement of livestock and other animal populations. This series seeks to showcase impactful contributions that deepen our understanding and application of genetic and genomic tools in breeding programs and welcomes demonstrations of success as well as theoretical proposals.

**Series Editors:**

- Emily Clark, The European Molecular Biology Laboratory's European Bioinformatics Institute (EMBL-EBI), United Kingdom
- Oscar Gonzalez-Recio, The University of Edinburgh, United Kingdom

The series will launch in Summer 2027, though articles will be published online shortly after acceptance via Advance Access. Papers will be collected and navigable across the 2 journals and promoted on social media and other venues in a manner similar to other series the journals have published.

Authors are invited to submit manuscripts by September 15, 2026, and should submit to their journal of choice. Manuscripts will be reviewed and edited through the standard peer review process and according to the usual standards of the journals. Some manuscripts submitted to GENETICS may be offered a transfer to G3: Genes|Genomes|Genetics or vice versa.

We encourage continued submissions past September 15, 2026, as we intend this series to be an ongoing and growing resource in this area. At submission, please choose the “Advances in Genetics and Genomics of Animal Breeding” article type in the submission system and mention the series in your cover letter.

Please share with your colleagues, and do not hesitate to contact any of the series editors or the GENETICS and G3: Genes|Genomes|Genetics Editorial Office with questions, suggestions, or presubmission inquiries.

